# Reduction of Time on the Ground Related to Real-Time Video Detection of Falls in Memory Care Facilities: Observational Study

**DOI:** 10.2196/17551

**Published:** 2021-06-17

**Authors:** Eleonore Bayen, Shirley Nickels, Glen Xiong, Julien Jacquemot, Raghav Subramaniam, Pulkit Agrawal, Raheema Hemraj, Alexandre Bayen, Bruce L Miller, George Netscher

**Affiliations:** 1 Department of Neuro-rehabilitation Hôpital Pitié-Salpêtrière Assistance Publique des Hôpitaux de Paris, Sorbonne Université Paris France; 2 Global Brain Health Institute Memory and Aging Center, Department of Neurology University of California San Francisco, CA United States; 3 SafelyYou, Inc San Francisco, CA United States; 4 Alzheimer’s Disease Center, Department of Neurology, University of California, Davis Sacramento, CA United States; 5 Computer Science and Artificial Intelligence Laboratory Massachusetts Institute of Technology Boston, CA United States; 6 Electrical Engineering and Computer Science Department University of California Berkeley, CA United States

**Keywords:** artificial intelligence, video monitoring, real-time video detection, fall, time on the ground, Alzheimer disease, dementia, memory care facilities

## Abstract

**Background:**

Lying on the floor for a long period of time has been described as a critical determinant of prognosis following a fall. In addition to fall-related injuries due to the trauma itself, prolonged immobilization on the floor results in a wide range of comorbidities and may double the risk of death in elderly. Thus, reducing the length of Time On the Ground (TOG) in fallers seems crucial in vulnerable individuals with cognitive disorders who cannot get up independently.

**Objective:**

This study aimed to examine the effect of a new technology called SafelyYou Guardian (SYG) on early post-fall care including reduction of Time Until staff Assistance (TUA) and TOG.

**Methods:**

SYG uses continuous video monitoring, artificial intelligence, secure networks, and customized computer applications to detect and notify caregivers about falls in real time while providing immediate access to video footage of falls. The present observational study was conducted in 6 California memory care facilities where SYG was installed in bedrooms of consenting residents and families. Fall events were video recorded over 10 months. During the baseline installation period (November 2017 to December 2017), SYG video captures of falls were not provided on a regular basis to facility staff review. During a second period (January 2018 to April 2018), video captures were delivered to facility staff on a regular weekly basis. During the third period (May 2018 to August 2018), real-time notification (RTN) of any fall was provided to facility staff. Two digital markers (TUA, TOG) were automatically measured and compared between the baseline period (first 2 months) and the RTN period (last 4 months). The total number of falls including those happening outside of the bedroom (such as common areas and bathrooms) was separately reported by facility staff.

**Results:**

A total of 436 falls were recorded in 66 participants suffering from Alzheimer disease or related dementias (mean age 87 years; minimum 65, maximum 104 years). Over 80% of the falls happened in bedrooms, with two-thirds occurring overnight (8 PM to 8 AM). While only 8.1% (22/272) of falls were scored as moderate or severe, fallers were not able to stand up alone in 97.6% (247/253) of the cases. Reductions of 28.3 (CI 19.6-37.1) minutes in TUA and 29.6 (CI 20.3-38.9) minutes in TOG were observed between the baseline and RTN periods. The proportion of fallers with TOG >1 hour fell from 31% (8/26; baseline) to zero events (RTN period). During the RTN period, 76.6% (108/141) of fallers received human staff assistance in less than 10 minutes, and 55.3% (78/141) of them spent less than 10 minutes on the ground.

**Conclusions:**

SYG technology is capable of reducing TOG and TUA while efficiently covering the area (bedroom) and time zone (nighttime) that are at highest risk. After 6 months of SYG monitoring, TOG was reduced by a factor of 3. The drastic reduction of TOG is likely to decrease secondary comorbid complications, improve post-fall prognosis, and reduce health care costs.

## Introduction

Falls are the leading cause of injuries among people aged 65 and older, with estimated yearly direct medical costs of US $637.2 million for fatal falls and US $31.3 billion for nonfatal falls in the United States [1]. Older adults with cognitive impairment have an increased risk of falling [2], and those with dementia living in nursing care facilities fall 4.1 times per year on average versus 2.3 times for other residents [3]. Additionally, individuals with dementia are the least likely to call for assistance when they cannot rise alone from the ground, and cognitive impairment is found to be the most significant factor that predicts lying on the floor for a long time after a fall [4].

Reducing the length of time on the floor after a fall is crucial because fall-related injuries include not only traumatic injuries associated with the acute fall but also comorbid complications related to prolonged post-fall immobilization. Artificial intelligence (AI) in the field of computer vision; innovative health technology using secure, network-attached storage; and a customized computer application together offer the potential to detect falls rapidly without the need for wearable devices and to support care in dementia care facilities [5]. In previous work using SafelyYou Guardian (SYG) technology [5], we showed that video monitoring of falls along with regular fall review by facility staff resulted in more accurate identification of falls, fall mechanisms, and the injuries related to the trauma itself (such as traumatic brain injury, for instance) [6]. More recently, we compared nonbeneficiaries to beneficiaries of SYG and found that review of fall footage enabled a significant reduction of visits to the emergency department, by 72% on average, due to better triaging of fall-related injuries [7]. In this article, we focus on subsequent length of time spent on the floor after falling as automatically measured by SYG technology in beneficiaries of SYG.

The objective here was to investigate the effect of the SYG real-time notification (RTN) system on early provision of post-fall care. We analyzed 2 lengths of time associated with a fall event: (1) The length of Time Until staff Assistance arrives (TUA) reflects the first support provided by professional caregivers, and (2) the length of Time On the Ground (TOG) in fallers is a prognostic marker reflecting risks for secondary post-fall complications in residents. Discrepancies between TUA and TOG are common. For example, a professional caregiver who arrives in a resident’s room shortly after a fall may wait for a second caregiver to come and assist the resident to his or her bed. These 2 digital care markers were measured over 10 months for consenting residents living in 6 memory care facilities. We hypothesized that introducing SYG would reduce TUA and TOG. Comparison of TUA and TOG markers between the baseline period and RTN period were computed to assess the effect of SYG technology on early post-fall care.

## Methods

### Design of the Study and Technology Equipment

**Figure 1:**
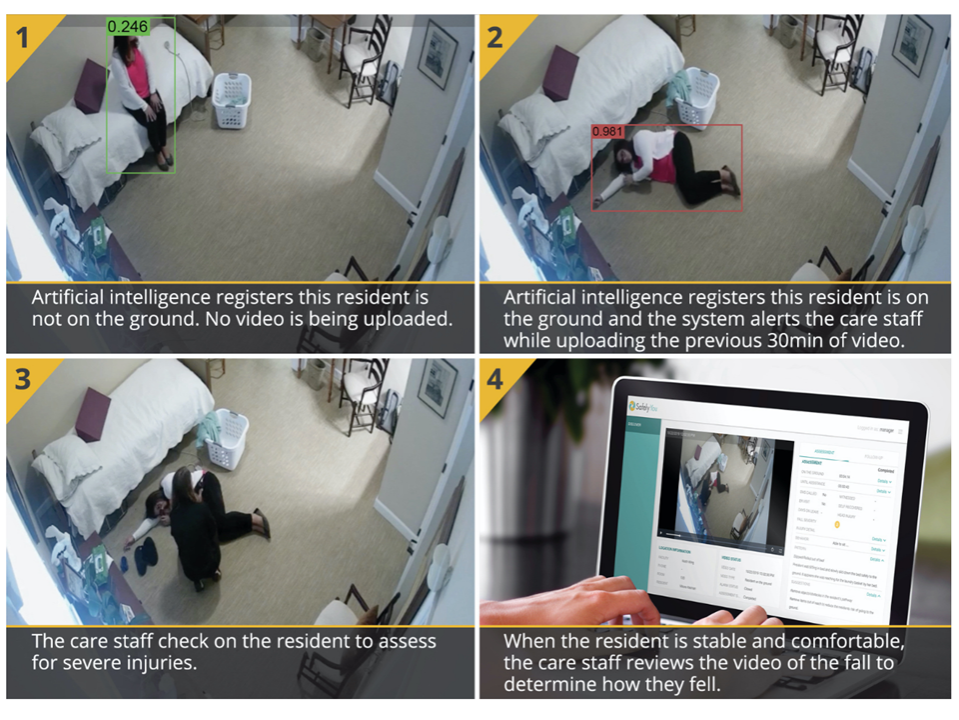
SafelyYou Guardian (SYG) technology provides automated notification of the fall, video footage (to assess fall severity), and care recommendations to facility staff.

This study is part of a larger project involving multilevel collaborations between engineers, researchers, clinicians, and care providers. SafelyYou develops computer vision algorithms (a subfield of AI focused on visual understanding) for automated, real-life, real-time detection and notification of falls in memory care facilities [5]. This observational study was carried out during 10 months in 6 memory care facilities in California, and a progressive introduction of SYG technology was carried out so that facility staff could get familiar with the technology. In each of the 6 facilities, wall-mounted cameras were installed in residents’ bedrooms of consenting elderly and families (at the exclusion of residents’ personal bathrooms, where no camera was placed) and in accordance with the privacy and ethical guidelines discussed below. There was no requirement about where the camera was placed, but cameras were generally placed in order to capture as much of the room as possible since the camera would not detect a fall that it could not see. The field of view of the camera was greater than 90 degrees such that an entire room could be captured if the camera was placed in an upper corner of the room. A fall was defined as an “unexpected event in which the participant comes to rest on the ground, floor, or lower level” [8]. A fall incident was detected by the AI-enabled camera system when a resident was identified on the floor, whatever his or her position (sitting or lying). This detection is independent of camera position, the number of individuals in the room, and which individual is on the floor. “On the floor” was defined to be when one body part other than the feet touched the floor, such as a knee, forearm, or the posterior. Falls were video captured 24 hours a day, 7 days a week, and video data were securely transmitted using a local network storage device. A secure, customized computer application gave facility staff access to the video footage and fall information of fall incidents (Figure 1).

**Figure 2:**
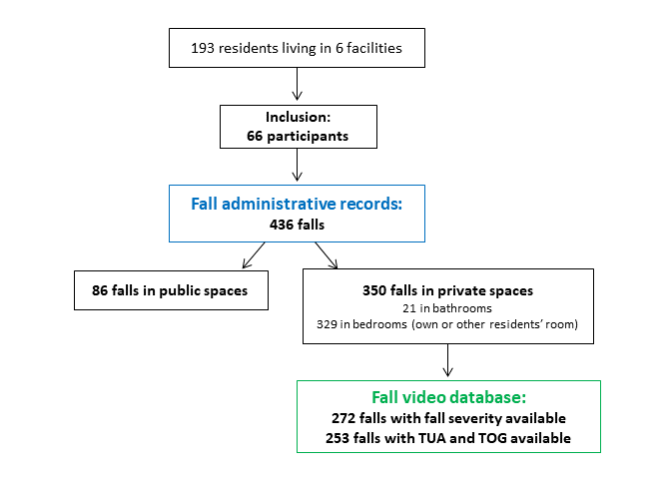
Flow chart showing inclusions and fall databases. TOG: Time On the Ground; TUA: Time Until staff Assistance arrives.

Between November 1, 2017 and August 31, 2018, falls were prospectively recorded in administrative reports and were video captured for consenting participants (Figure 2). The study entailed 3 successive periods over 10 months. During the 2-month baseline installation period (November 2017 and December 2017), a set of control measures was performed following setup of the cameras and SYG video captured falls, but video footage was not provided on a regular basis to facility staff review. During a second 4-month period (January 2018 through April 2018), video captures of falls were delivered to facility staff on a weekly basis so that they could get familiar with the technology and footage review; however, RTN directly occurring after a fall was not activated. Finally, during the third 4-month period (from May 2018 through August 2018), RTN of any fall was provided to facility staff via a phone call so that the faller could receive immediate assistance after the alarm onset.

### Outcome Measures

For each participant, the number of falls occurring in private and public spaces of the memory care facility was recorded by facility staff over the 10-month period: Private spaces refer to resident’s personal areas (ie, individual’s bedroom and bathroom), while public spaces refer to common areas shared by residents (eg, dining rooms, hallways, lounges). Falls happening in bathrooms, in nonparticipants’ bedrooms, and in common spaces of the memory care facilities were not video recorded but were reported through existing incident reporting processes.

For participants only, a video review allowed the classification of falls according to their severity. A fall was classified as a “behavioral fall” when the resident showed an intentional slow descent with safe landing but no recovery to prior position. Other falls were granted a severity score using the 4-point Hopkins Falls Grading Scale [9]. This scale stratifies fall severity in near fall (Grade 1), minor fall with no need for medical attention (Grade 2), moderate fall requiring medical attention (Grade 3), and severe fall requiring hospital admission (Grade 4).

Two care markers were automatically generated by the SYG technology in order to assess early provision of post-fall care. TUA timed the delay between the detection of the fall and the detection of staff arrival in the resident’s bedroom. TOG timed the delay between the detection of the fall and the resident’s return to a comfortable position with assistance from facility staff. Measurement of these 2 digital markers allowed classification of falls according to their duration [4] (less than 10 minutes, 10 to 30 minutes, 30 to 60 minutes, and more than 60 minutes).

### Ethical Procedures and Privacy

Privacy and consent procedures were developed with support from the Institutional Review Board of the University of California, Berkeley [10] and in collaboration with the California Department of Social Services Community Care Licensing Division. The Committee for Protection of Human Subjects of the University of California, Berkeley approved the study protocol. Procedures have been published in detail [6].

Residents living within the care facilities showed severe cognitive impairment related to Alzheimer disease and related dementias. As a consequence, surrogate consent by a legally authorized representative (usually next of kin) was required for the present study. The legally authorized representatives of the residents were given oral and written detailed information about the study and were provided the informed consent document. The study was explained to the affected individuals living in the facility, and if they provided any verbal or nonverbal indication that they did not wish to have the cameras in their bedroom, they were not included. The legally authorized representative was the person who could say yes to the study, thus providing informed consent, but the resident retained the right to decline the study at any time, thus providing assent. Cameras were located high in a corner in the bedroom, but not in the bathroom, and remained visible to the participants. Camera audio recording was disabled. Signs were posted visibly on the door of each bedroom in which video recording occurred as a reminder to residents, families, and facility staff that participants were being video monitored in the bedrooms.

### Statistical Analyses

TUA and TOG variables were quantified in minutes and are described with means, median, standard deviation, minimum, and maximum. Comparison of means of TUA and of TOG between the baseline period and the RTN period was performed with nonparametric Wilcoxon rank-sum tests because of nonnormality of the data. A one-way analysis of variance (ANOVA) was conducted to determine if TUA differed between facilities. The TUA amounted to 0 minutes when a facility staff was already in the bedroom witnessing the live fall. Falls with a TUA equal to 0 were counted over the 3 periods, and Wilcoxon rank-sum comparison tests were performed with and without falls with TUA equal to 0. Statistical analyses were performed using Stata version 15 (Stata Corp; College Station, Texas) and R (R Programming).

## Results

A total of 66 individuals out of 193 residents (34.2%) living in the 6 facilities at the time of inclusion participated in the study. These 66 participants suffered from Alzheimer disease and related dementias. They had a mean age of 87 (SD 6.9; minimum 65, maximum 104) years and were nearly two-thirds women (42/66, 64%). The majority of the participants (54/66, 82%) were determined to be recurrent fallers with an average of 6.6 (SD 8.6; minimum 1, maximum 49) falls per individual over the 10-month period. No difference in the number of falls per faller was found according to gender.

**Figure 3:**
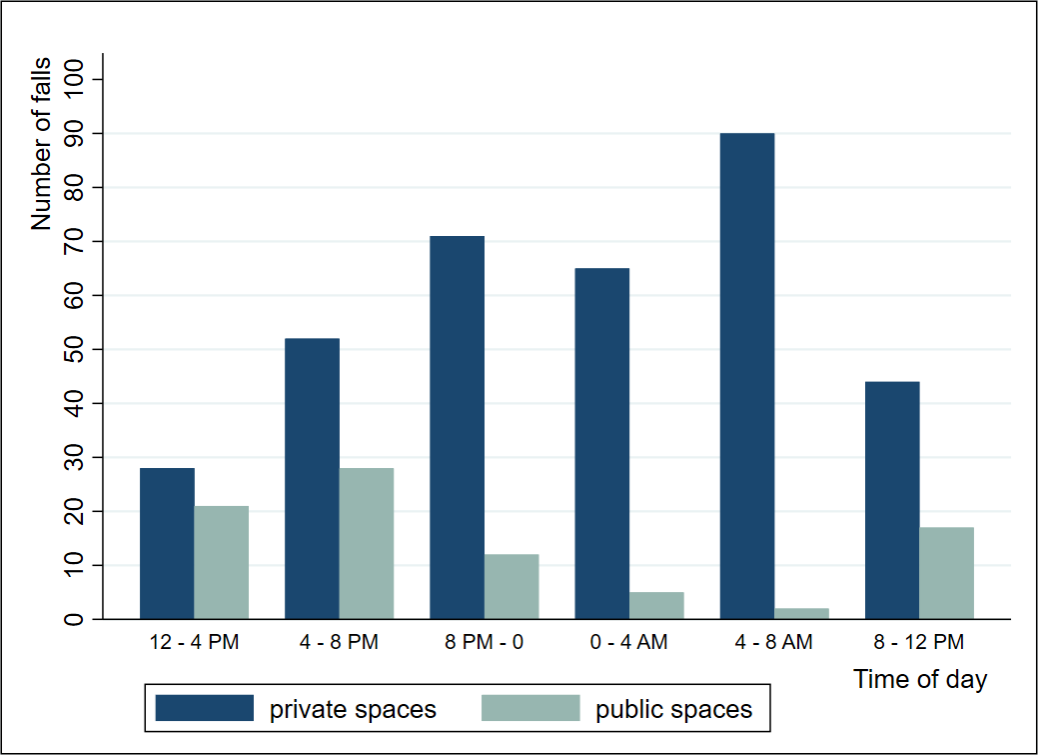
Display of falls according to location and day schedule. Private spaces refer to residents' personal areas (ie, individual’s bedroom and bathroom); public spaces refer to common areas shared by residents (eg, dining rooms, hallways, lounges).

A total of 436 falls were reported in administrative records of those 66 study participants over 10 months. Among these falls, 86 (86/436, 19.7%) occurred in common areas, and 350 (350/436, 80.3%) occurred in private spaces (including falls in participants’ personal bedrooms, in participants’ personal bathrooms, and in bedrooms of other nonparticipant residents). As displayed in Figure 3, 64.6% (226/350) of falls that happened in private spaces occurred during nighttime (8 pm to 8 am), with a maximal spike of incidents between 4 am and 8 am. Video data about fall severity were fully available for 272 events, including a total of 93 falls (93/272, 34.2%) classified as “behavioral falls with safe landing.” Grading of the severity of the remaining falls showed 18 (18/272, 6.6%) near falls with independent recovery, 139 (139/272, 51.1%) minor falls, and 22 (22/272, 8.1%) moderate and severe falls (18 and 4, respectively).

Video data about TUA and TOG markers were fully available in 253 falls (missing videos and gaps in video are attributed to technical issues such as gaps in WiFi connectivity). Both TUA and TOG showed a wide variation ranging from zero (or a few seconds) to more than 3.5 hours (maximum TUA: 214.2 minutes; maximum TOG: 215 minutes; both during the 2 first periods before RTN). The one-way ANOVA showed that there was a statistically significant difference in TUA between facilities (F_5_=3.79, *P*=.003). We found that TUA was higher in the standalone memory care facilities (2 of the 6 facilities) than in the other 4 facilities (ie, memory care units within larger assisted-living facilities; Wilcoxon rank-sum test *P*=.002). The difference between TOG and TUA over 10 months was similar over the 3 time periods (7.8 minutes at baseline, 6.6 minutes during weekly review, and 6.9 minutes during the RTN period) and often related to the need for more than one health care professional to help lift fallers. In 2.4% (6/253) of falls, residents got up independently before staff arrival, resulting in a larger TUA than TOG. A facility staff member was already in the bedroom during the fall event for 17.4% (44/253) of cases, resulting in a TUA of 0 minutes.

The TUA amounted to an average 35.3 (SD 48.3, median 7.6) minutes, 15.9 (SD 32.1, median 2.4) minutes, and 7.0 (SD 9.5, median 2.8) minutes during the baseline, weekly review, and RTN periods, respectively. TUA differed significantly between the baseline period (November 2017 and December 2017) and the RTN period (May 2018 to August 2018; *P*=.004), with a mean reduction in TUA of 28.3 (CI 19.6-37.1) minutes. The TOG amounted to an average 43.2 (SD 48.8, median 23.3) minutes, 22.9 (SD 32.9, median 11.5) minutes, and 13.6 (SD 12.1, median 9.3) minutes during the baseline, weekly review, and RTN periods, respectively. TOG differed significantly between the baseline period and RTN period (*P*=.043), with a mean reduction in TOG of 29.6 (CI 20.3-38.9) minutes. We found similar results after removing those falls with a TUA equal to 0 minutes (ie, a significant difference in TUA between the baseline period and RTN period [*P*=.004], with a mean reduction in TUA of 37.7 [CI 27.8-47.7] minutes).

**Figure 4:**
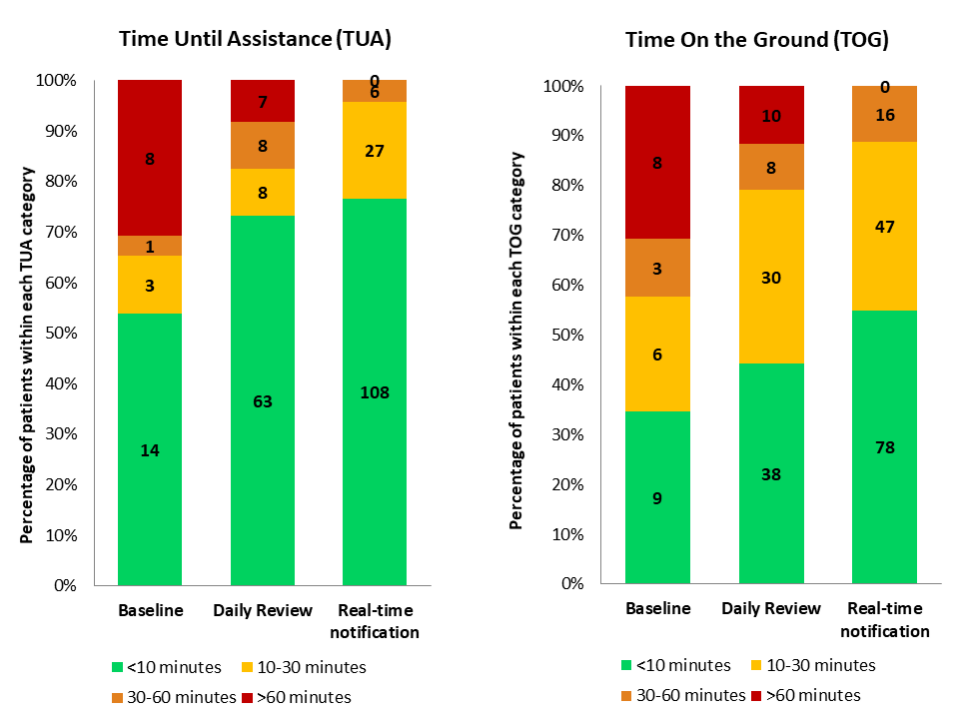
Proportion of falls with short, medium, and long (A) Time Until staff Assistance arrives (TUA) and (B) Time On the Ground (TOG) measures during the 3 time periods.

As shown in Figure 4, there was an increase in the proportion of individuals lying on the floor for less than 10 minutes and a related decrease in the proportion of individuals experiencing a TOG of over 60 minutes, with no such case during the RTN period. During the RTN period, 76.6% (108/141) of fallers received human assistance in less than 10 minutes and 55.3% (78/141) of them spent less than 10 minutes on the ground. In parallel, there was a drastic drop from 31% (8/26) to zero cases of those individuals who experienced a TOG of more than 1 hour (Figure 4).

## Discussion

The present observational study provides novel insight about real-life falls and early post-fall care as captured through continuous video monitoring, AI detection, and notification of falls in bedrooms in memory care facility settings. We found that a significant reduction (0.5 hour) in TUA and TOG was associated with video monitoring and RTN of falls. During RTN, three-quarters of fallers received staff assistance in less than 10 minutes, and half of them recovered to a comfortable position in less than 10 minutes, while the proportion of very long TOG (ie, more than 60 minutes) was reduced. These results suggest that SYG technology was able to reduce a crucial severity marker of falls (ie, prolonged immobilization on the ground) that is usually associated with medical complications and poor post-fall prognosis.

The study provides ecological data of falls happening in memory care facilities using both administrative records and video monitoring reports. Regarding locations of falls, over 80% of the fall incidents happened in private spaces (mostly residents’ bedrooms) confirming a similar finding that 75% of falls happen in bedrooms, as estimated from 70,000 fall incidents recorded administratively in 528 long-term care facilities [11]. Since residents are usually alone when they are in their bedrooms (as opposed to common areas where more facility staff can assist them), it appears necessary to place fall detector systems like SYG primarily in these high-risk areas to ensure continuous fall monitoring [4]. Regarding time of the day when residents fall, we found a high occurrence during nighttime (when there are fewer facility staff and residents spend more time on their own in their rooms), suggesting potential for adapting schedules and safety rounds in order to minimize risk of falls. Indeed, sleep disturbances (such as reduced sleep time, sleep fragmentation, and nocturnal wandering [12]), impaired circadian rhythmicity with sundowning [13], nocturnal effects of drugs [14], and urination disorders [15] are common in dementia and yield an augmented risk of night falls. Regarding the severity of falls, our results parallel previous research reporting that the vast majority of falls are classified as minor falls and do not include traumatic injuries [16,17]. In turn, this result points out the benefit of reviewing video footage in order to better screen injuries and avoid unnecessary referral to the emergency department and unnecessary health care costs [7]. The review also allows identification of the mechanisms of falls [18]. In long-term care facilities, most falls were related to self-induced weight shifting (with an equal frequency during walking, transferring, and standing) that can further lead to more balance assessment and fall prevention [18].

The study provides quantitative post-fall measures (TUA, TOG) that were automatically measured with an AI-driven technology. To our knowledge, our group is one of the first to report these automatically derived time measures. By contrast, research in the field relies primarily on surveys and retrospective interviews of patients and proxies, leading to recall bias [19] and estimation errors [4,13,16,20]. Our baseline result of 30.8% of residents lying on the floor for more than 1 hour (TOG) replicates a similar proportion of 30% stemming from a large survey of 265 falls from a mixed population of home-dwelling and institutionalized elderly people over 90 years old [4]. For the first time, we were able to show that this major and frequent risk of long TOG events could be successfully modified in just a few months and was reduced down to zero events. We found a TUA logically lower than TOG in more than 93% of the cases; as previously reported, 80% of vulnerable individuals are unable to get up without help, and getting up from the floor often requires more than one health professional [4]. The same study also showed that 94% of institutionalized elderly who could not get up alone and who had access to a call alarm did not activate the alarm system to summon help [4]. In geriatric patients visiting the emergency department after a fall, only 16% of fallers used their personal emergency response system to call for assistance [20]. Our results highlight how AI fall detector systems like SYG do not require a faller’s direct action to activate staff assistance [20], which is necessary to deliver a critical care need and enhance safety for vulnerable individuals. Variations in TUA might be related to different facility protocols and staff availability, and caregivers also show diverse experience and training. In turn, facility staff began to incorporate the video review in their care practice after an adoption period (accounting for a reduction of TUA during the second period), and policy changes might also be observed (such as additional safety rounds for residents at high risk and environmental changes as previously documented [6]).

TUA and TOG care markers showed an average 29-minute decrease, indicating that much faster assistance to help fallers recover to a comfortable position could be provided, instead of relying on regular care rounds only. There is extensive literature about the additional critical comorbidities that are related to the long lie [21] and inability to get up [22], including metabolic and physical consequences (dehydration, hypothermia, rhabdomyolysis, renal failure, pressure ulcers [23]), psychological complications (post-fall syndrome with fear of falling again [24] and activity limitation [16]), higher risk for recurrent falls [4], and loss of autonomy [16]. Lying on the floor after a fall for a long period of time was also found to nearly double the risk of death in elderly adults [23]. On average, the RTN resulted here in assigning a “fall incident” to staff as a red flag priority among their other ongoing care tasks, accounting for the substantial reduction in TUA and in TOG.

TOG has been long considered a core severity factor of falls as well as a predictor of post-fall prognosis [22], while it has suffered from quantification weaknesses at the same time [4]. We demonstrate here that AI technology can overcome this issue and can now generate a robust and reliable care marker. In turn, shorter TUA could be considered a quality-of-care criterion for patients, families, and care providers, while TOG could be integrated more systematically in falls reporting. Taking into account that fall guidelines and management entail standardization needs [8,25-27], our contribution to the field is to provide evidence for the feasibility and usefulness to integrate these digital care markers into dementia care practice. Other strategies in the field of fall detection use different technology including wearable alert systems [28] (which require residents and staff to remember to wear and reload the device regularly), accelerometer-based fall detection [29], and nonwearable fall detection systems [30] (either based on radar and optical sensors or that use fall mats and bed alarms). As SYG, these emerging technologies warrant further controlled trials in order to assess and compare industrial responses to fall detection needs and their associated costs.

This study has some limitations. All residents were not included: Residents or their legally authorized representatives who did not opt in cited privacy concerns of video recording or that their relative was not yet at fall risk in need of the technology. Regarding variations in TUA, we were able to observe that standalone facilities had higher TUA than memory care units based within larger assisted living facilities, but other unreported factors might account for slower assistance received by fallers. We did not evaluate the cognitive-behavioral disorders in fallers with a formal cognitive assessment, and rates and types of comorbidities related to the long TOG were not measured. In the near future, implementation of randomized trials is needed to compare SYG use versus no use on the potential differences associated with diverse levels of cognitive-behavioral impairment and secondary comorbid outcomes related to falls in parallel to TUA or TOG outcomes. Cost-effectiveness analyses of interventions involving new fall detectors are needed in the field: an economic evaluation would compare costs, including those associated with the new technology and those reduced because unnecessary referrals to the emergency department were avoided.

To conclude, SYG focuses on unmet care needs and efficient new strategies in memory care facilities while enhancing safety in the bedroom and during time zones that represent the highest risk of falls. Unlike other nonmodifiable risk factors in falls [31], TOG can be significantly reduced to improve post-fall prognosis. Automated quantification of TUA and TOG can complement current care practice and support better quality of care for vulnerable individuals. In turn, reducing TOG has the potential to decrease future morbidity, unnecessary referral to the emergency department, and related costs [4,7].

## Abbreviations

AI: artificial intelligence
ANOVA: analysis of variance
RTN: real-time notification
SYG: SafelyYou Guardian
TOG: Time On the Ground
TUA: Time Until staff Assistance arrives
